# Induced Suppression of the Left Dorsolateral Prefrontal Cortex Favorably Changes Interhemispheric Communication During Bimanual Coordination in Older Adults–A Neuronavigated rTMS Study

**DOI:** 10.3389/fnagi.2020.00149

**Published:** 2020-05-26

**Authors:** Stefanie Verstraelen, Kim van Dun, Julie Duque, Hakuei Fujiyama, Oron Levin, Stephan P. Swinnen, Koen Cuypers, Raf L. J. Meesen

**Affiliations:** ^1^Neuroplasticity and Movement Control Research Group, Rehabilitation Research Institute (REVAL), Hasselt University, Diepenbeek, Belgium; ^2^Institute of Neuroscience, Université Catholique de Louvain, Louvain-la-Neuve, Belgium; ^3^Discipline of Psychology, Exercise Science, Chiropractic and Counselling College of Science, Health, Engineering and Education, Murdoch University, Murdoch, WA, Australia; ^4^Movement Control and Neuroplasticity Research Group, Department of Movement Sciences, Group Biomedical Sciences, KU Leuven, Leuven, Belgium; ^5^Leuven Brain Institute (LBI), KU Leuven, Leuven, Belgium

**Keywords:** aging, bimanual coordination, dorsolateral prefrontal cortex, interhemispheric interaction, repetitive transcranial magnetic stimulation

## Abstract

Recent transcranial magnetic stimulation (TMS) research indicated that the ability of the dorsolateral prefrontal cortex (DLPFC) to disinhibit the contralateral primary motor cortex (M1) during motor preparation is an important predictor for bimanual motor performance in both young and older healthy adults. However, this DLPFC-M1 disinhibition is reduced in older adults. Here, we transiently suppressed left DLPFC using repetitive TMS (rTMS) during a cyclical bimanual task and investigated the effect of left DLPFC suppression: (1) on the projection from left DLPFC to the contralateral M1; and (2) on motor performance in 21 young (mean age ± SD = 21.57 ± 1.83) and 20 older (mean age ± SD = 69.05 ± 4.48) healthy adults. As predicted, without rTMS, older adults showed compromised DLPFC-M1 disinhibition as compared to younger adults and less preparatory DLPFC-M1 disinhibition was related to less accurate performance, irrespective of age. Notably, rTMS-induced DLPFC suppression restored DLPFC-M1 disinhibition in older adults and improved performance accuracy right after the local suppression in both age groups. However, the rTMS-induced gain in disinhibition was not correlated with the gain in performance. In sum, this novel rTMS approach advanced our mechanistic understanding of how left DLPFC regulates right M1 and allowed us to establish the causal role of left DLPFC in bimanual coordination.

## Introduction

Bimanual coordination is essential in many daily tasks, such as tying shoelaces or driving a car. Age-related declines in bimanual coordination have been widely reported (Stelmach et al., 1988; Swinnen, 1998; Wishart et al., 2000; Temprado et al., 2009; Bangert et al., 2010; Summers et al., 2010; Fling et al., 2011; Lin et al., 2014; Serbruyns et al., 2015a,b; Leinen et al., 2016; King et al., 2018). Because altered bimanual skills in daily life affect functional independence in older adults, research towards a better understanding of the neural correlates underlying bimanual coordination is highly relevant (Maes et al., 2017).

When performing cyclical inter-limb coordination tasks, both young and older adults recruit areas involved in executive functions, such as the dorsolateral prefrontal cortex (DLPFC). This recruitment is most prominent during the initial learning period (Puttemans et al., 2005; Beets et al., 2015; Santos Monteiro et al., 2017), and is more pronounced in older adults as compared to younger adults (Heuninckx et al., 2005, 2008; Goble et al., 2010; Santos Monteiro et al., 2017). Furthermore, age-related alterations in neural processes not only occur during movement execution but also during planning (Sterr and Dean, 2008; Levin et al., 2011; Berchicci et al., 2012; Cuypers et al., 2013; Duque et al., 2016; Santos Monteiro et al., 2017). As preparatory neural activity predicts subsequent motor performance (Churchland, 2015; Michaels et al., 2015), research into neural mechanisms regarding the preparation and execution of bimanual coordination can offer profound insights into how DLPFC affects motor behavior in older adults. Notably, even though there is mounting evidence for DLPFC involvement during bimanual coordination, it remains unclear how this is causally related to motor performance. Therefore, the causal role of DLPFC in supporting motor performance requires further validation.

Recent dual-coil transcranial magnetic stimulation (TMS) research of Fujiyama et al. (2016b), using a conditioning stimulus (CS) on DLPFC and a test stimulus (TS) on the primary motor cortex (M1), indicated that the magnitude of the release of interhemispheric DLPFC-M1 inhibition (i.e., disinhibition) during motor planning of a complex bimanual task was significantly correlated with better performance. This finding was irrespective of age, but in older adults, the interhemispheric DLPFC-M1 disinhibition was reduced. Here, we used repetitive TMS (rTMS) to substantiate and complement previous correlational evidence by directly manipulating brain function. Specifically, short-train rTMS was used, which is known to transiently inhibit local excitability for ~500–1,000 ms (Modugno et al., 2001; Duque et al., 2012), inducing a “virtual” lesion in DLPFC (see also Davare et al., 2006; Duque et al., 2010). The effect of transiently inhibiting (i.e., suppressing) DLPFC was measured: (1) on interhemispheric connectivity between DLPFC and M1 by using a dual-coil TMS method (Ferbert et al., 1992; Gerloff et al., 1998; Chen et al., 2003); and (2) on behavioral characteristics of bimanual motor performance. To investigate how DLPFC regulates M1 through interhemispheric interaction (IHI), we compared results of a standard dual-coil paradigm as in Fujiyama et al. (2016b) with those of the more innovative paradigm, in which short-train rTMS over DLPFC preceded the CS on DLPFC to modulate IHI. We predicted that local suppression of DLPFC with rTMS would release DLPFC-M1 inhibition, thereby confirming that the DLPFC-M1 interaction reflects disinhibition (i.e., inhibition of interhemispheric inhibition), rather than facilitation. Thus, for older adults, we expected that rTMS would “restore” the DLPFC-M1 disinhibition (Hypothesis 1). Concerning the behavioral effect, we hypothesized that suppressing DLPFC in older adults would, therefore, improve bimanual motor performance (Hypothesis 2).

## Materials and Methods

### Participants

For sample size calculation, we used the GPower software (version 3.1.9.4). To detect medium effect sizes (*f* = 0.25) in the omnibus test for IHI modulation, considering the factors of age, rTMS, and time point (baseline vs. preparation, see below for details) and their interactions, we set *α* = 0.05 and power = 0.80. The power analysis indicated that the current study required at least 34 participants. To anticipate possible drop-outs, we argued that an additional 20% (i.e., seven participants, total *n* = 41) would be sufficient. Also, note that the effect sizes of previous studies using similar protocols (Duque et al., 2012; Fujiyama et al., 2016b) were larger than 0.25 (*f* = ~0.5).

A total of 51 healthy participants aged between 18–30 and 60–75 years were initially recruited for this study. Participants had a normal or corrected-to-normal vision; they were free of any musculoskeletal disorders in the hands that could interfere with bimanual task performance [see “Bimanual Tracking Task (BTT)” section]; they reported no history of neurological or psychiatric disorders, and had not played a musical instrument for at least 3 years. Participants were excluded if they were left-handed [as assessed with the Edinburgh Handedness Questionnaire (Oldfield, 1971)], or if they did not meet the safety criteria for Magnetic Resonance Imaging (MRI) and TMS, based on standard screening questionnaires of the University Hospital Gasthuisberg (Leuven) and TMS guidelines by Rossi et al. (2009), respectively. Of the 51 participants that met the preliminary inclusion and exclusion criteria, 10 were excluded because they had a resting motor threshold (rMT) higher than 50% of maximum stimulator output. This particular exclusion criterion was applied to ensure the comfort of participants during the experiment because trains of rTMS on DLPFC at high intensities can be uncomfortable due to the activation of muscular tissue.

Hence, a total of 41 healthy participants (21 young, 20 older adults) were included in this study. The young adults had an age ranging from 18 to 26 years (mean ± SD = 21.57 ± 1.83; 11 females) and the older adults had an age ranging from 62 to 75 years (mean ± SD = 69.05 ± 4.48; 10 females). Scores on the Edinburgh Handedness Questionnaire (Oldfield, 1971) ranged from +40 to +100 (mean ± SD = +94.65 ± 12.29), indicating that all participants were right-handed. Older adults were additionally screened for mild cognitive impairments using the Montreal Cognitive Assessment questionnaire (Nasreddine et al., 2005). A cut-off score of 23 was used as this shows better diagnostic accuracy as compared to the conventional cut-off score of 26, when applied in healthy older adults (Carson et al., 2018). Participants had scores within the normal range (mean ± SD = 27.15 ± 1.95).

All participants provided written informed consent before participation and were financially compensated. The protocol was conducted following the Declaration of Helsinki (1964) and was approved by the local ethical committee of the University Hospital Gasthuisberg (Leuven) in Belgium (S60448).

### Bimanual Tracking Task (BTT)

**Figure 1:**
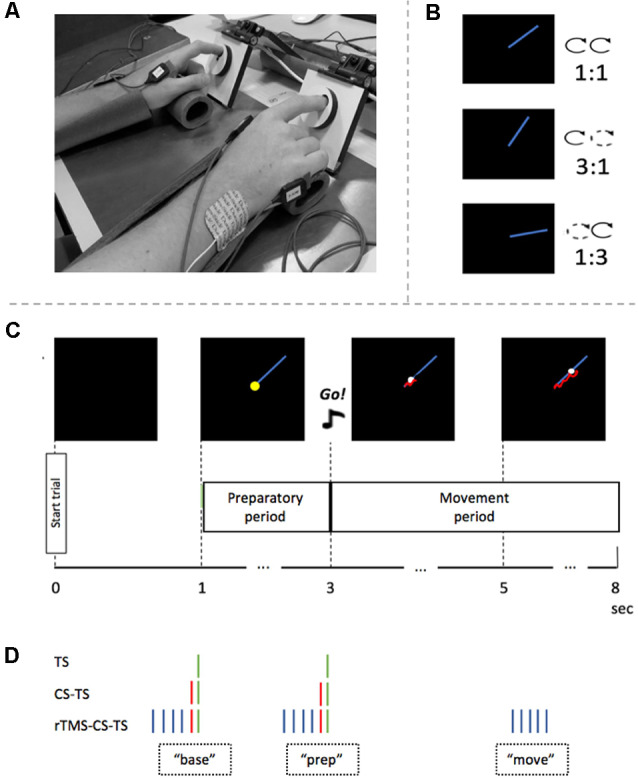
Experimental set-up and procedure. **(A)** Experimental setup. Arms were placed in palm rests for comfort. Index fingers were placed in two rotatable dials during the task. **(B)** Three different task conditions and their inter-hand frequency ratios. **(C)** Timeline of a single bimanual tracking task (BTT) trial. After 1 s, the preparatory period started by showing the blue target line. Two seconds later, an auditory “Go”-signal indicated that the participant had to start rotating the dials bimanually to follow the white target dot. Feedback of actual performance is visualized by a red tail-like line. **(D)** Four TMS conditions and their timing in a trial. A green stripe represents the TS on right primary motor cortex (M1). A red stripe represents the CS on the left dorsolateral prefrontal cortex (DLPFC). The blue stripes represent the rTMS train on the left DLPFC. Abbreviations: base, baseline measure at the time of target template onset; prep, measure in the preparatory period; move, measure in the movement period; TS, Test Stimulus; CS, Conditioning Stimulus; rTMS, repetitive TMS.

A bimanual visuomotor tracking task (BTT) was used (Sisti et al., 2011). The BTT setup was optimized for TMS experiments targeting the First Dorsal Interosseous (FDI) muscle (Fujiyama et al., 2016a, b). Participants were seated on a chair, with their arms pronated on a table in front of them. A computer screen was placed in front of the participant at a distance of ~75 cm. Each index finger was placed in a small groove (diameter of 1.5 cm) which was carved out on a rotatable dial and palm rests were used for comfort (Figure 1A). The goal of the BTT was to manipulate the position of the cursor on the computer screen by bimanually rotating the two dials to follow a moving target dot on a straight line as accurately as possible. Left and right dial rotations were associated with cursor movement along the ordinate and abscissa, respectively.

A BTT trial started with a black square of 16 × 16 cm on the computer screen (Figure 1C). After 1 s, a blue straight target line of 7 cm appeared in the right upper quadrant of the black square and indicated the start of the preparatory period. The target dot was situated on the left end of the line. Two seconds later, an auditory, imperative Go-signal was presented for 500 ms, which announced the end of the preparatory period and the start of the movement period. At the same time, the target dot started to move along the target line at a constant speed. The participant was instructed to follow the target dot with the cursor as accurately as possible by rotating the two dials in a clockwise manner (Figures 1B,C). Three task conditions differed in relative inter-hand frequencies (1:1, 1:3, 3:1). While the inter-hand frequency was the same for the 1:1 task condition, both index fingers had to move at different speeds during the 3:1 and 1:3 task conditions. In the 3:1 condition, the left index had to rotate the dial three times faster than the right index, while in the 1:3 condition the opposite pattern was required (Figure 1B). Hands were covered so that the participant could not visually direct his/her hand movements. Instead, he/she received on-line instantaneous visual feedback of the track of the cursor, by means of a red line. A full trial had a duration of 8 s, with an inter-trial interval of 2 s (Figure 1C).

**Figure 2:**
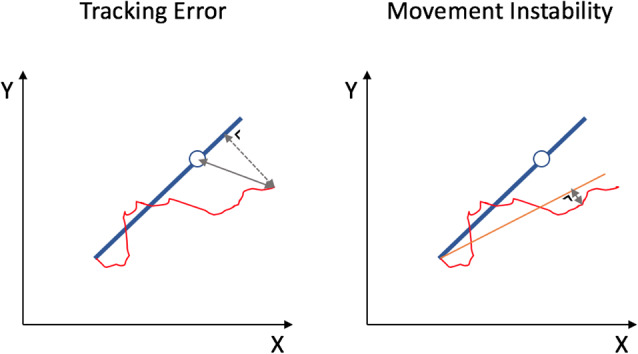
Two outcome measures for BTT performance. Tracking Error is the sum of the Euclidean distance between the subject’s cursor and the target dot (full line) plus the shortest distance between the subject’s cursor to the blue target line (dashed line), measured in arbitrary units and averaged throughout the trajectory. Movement Instability is measured as the shortest distance between the subject’s cursor and subject’s mean track (orange line), averaged throughout the trajectory.

The x and y coordinates of the target dot and the subject’s cursor were sampled at 100 Hz. The performance was assessed by two outcome measures: Tracking Error (TE) and Movement Instability (MI; Figure 2). TE is a measure for track accuracy, which allows considering both the speed of the movement and deviations of the cursor from the target line. Specifically, TE is the sum of the Euclidean distance between the subject’s cursor and the target dot plus the orthogonal distance between the subject’s cursor to the blue target line, measured in arbitrary units and averaged throughout the trajectory. In contrast, MI reflects the stability (smoothness) of the bimanual movement, regardless of the speed or target coordination pattern, and is calculated by the shortest distance between the subject’s cursor and subject’s mean track (i.e., the fitted line through the cursor’s trajectory), averaged throughout the trajectory. Performance outcomes were processed offline using Matlab (2018a, The MathWorks Inc., USA).

### Transcranial Magnetic Stimulation (TMS) and Electromyographic Recording

#### TMS Conditions and Outcome Measures

In the current study, we focused the left DLPFC—right M1 interaction, because the suppression of motor-evoked potentials (MEPs) during movement preparation is generally more pronounced in the non-dominant hand (Leocani et al., 2000; Duque et al., 2007).

Three different TMS conditions could be applied at two different time points during motor preparation, and a fourth condition could be applied during movement (see Figure 1D and section “Experimental Protocol” for a detailed description of these time points).

In the first TMS condition, a single TS was applied over the right M1, which allowed to measure corticospinal excitability. Corticospinal excitability was assessed by calculating the average peak-to-peak MEP amplitude in the left FDI muscle within a time window of 10–80 ms following the TS. In the second TMS condition, this TS was preceded by a single CS over left DLPFC with an inter-stimulus interval of 60 ms (Ni et al., 2009). This dual-pulse (CS-TS) condition was used to assess the IHI between left DLPFC and right M1. IHI was determined as the average MEP amplitude in the left FDI in CS-TS trials, relative to the average MEP amplitude in response to a single TS (i.e., IHI = MEP_(CS-TS)_/MEP_TS_). In the third condition, the CS-TS pulses were preceded by four repetitive pulses (rTMS) over left DLPFC, resulting in a total of five (4 rTMS + CS) pulses over DLPFC applied at 10 Hz. This short-train rTMS application is considered to perturb and to suppress ongoing activity in the underlying cortex (Davare et al., 2006; Duque et al., 2010) for at least 500 ms (Modugno et al., 2001; Duque et al., 2012). This rTMS-CS-TS condition served to study the effect of a transient virtual lesion in the left DLPFC on DLPFC-M1 IHI, expressed as “IHI_LESION_.” IHI_LESION_ was determined as the average MEP amplitude in the left FDI in rTMS-CS-TS trials, relative to the average MEP amplitude in response to a single TS (i.e., IHI_LESION_ = MEP_(rTMS-CS-TS)_/MEP_TS_).

In the fourth TMS condition, the transient perturbation by rTMS was applied during the execution of the bimanual movement. More specifically, five pulses were given over left DLPFC at 10 Hz 2 s after the imperative Go-signal (Figure 1D). This rTMS_move_ condition was implemented to assess the influence of DLPFC suppression on ongoing bimanual coordinative activity, expressed by TE and MI.

#### Neuronavigation and TMS Settings

Before the experiment, the DLPFC localization was individually determined on a 3D brain reconstruction rendered by the neuronavigation system (Brainsight, Rogue Research Inc., Montreal, QC, Canada), based on a structural T1-weighted image obtained from each participant (Philips Ingenia 3 Tesla, MPRAGE, TR/TE = 9.6 ms/4.6 ms, voxel size = 0.98 mm × 0.98 mm × 1.2 mm, the field of view = 250 mm × 250 mm × 240 mm, 200 sagittal slices). To localize DLPFC, we used the practical algorithm described by Mylius et al. (2013). First, the boundaries of the middle frontal gyrus were established. The anterior border was determined by the anterior termination of the olfactory sulcus, while the superior and inferior frontal sulci were marked as the upper and lower boundaries of the middle frontal gyrus, respectively. Lastly, the posterior border was drawn by the precentral sulcus, joining the respective intersections of the superior and inferior frontal sulci. When the middle frontal gyrus was defined, this structure was divided into three equal parts. DLPFC was located in the middle of the separating line between the anterior and central thirds of the middle frontal gyrus at the cortical surface. Mean Talairach coordinates of the DLPFC are shown for both young and older participants in Table 1.

**Table 1:** Mean dorsolateral prefrontal cortex (DLPFC) Talairach coordinates per age group (Mean ± Standard Deviation).

Younger			Older		
*x*	*y*	*z*	*x*	*y*	*z*
−34.83 ± 4.06	30.92 ± 4.80	38.32 ± 4.41	−34.44 ± 3.65	33.37 ± 3.20	32.73 ± 3.38

For rTMS and CS application, an MCF-B70 static cooled 97 mm figure-8 coil (Magventure, Denmark) was held perpendicular to the mid-sagittal line over the left DLPFC (Ni et al., 2009). All pulses on DLPFC (i.e., rTMS-train and CS) were biphasic (pulse width of 280 μs), and the intensity was set at 120% of the individual rMT of the left M1. The rMT is defined as the minimal stimulation intensity required to evoke MEPs with a peak-to-peak amplitude >50 μV in at least five out of 10 consecutive trials. For the TS (monophasic) over right M1, a 70 mm figure-8 coil, connected to a Magstim 200 (Magstim Company, Whitland, UK), was used to target the motor hotspot of the left FDI. The handle of the Magstim coil was oriented with an angle of 45 degrees away from the mid-sagittal line. The intensity of the TS on right M1 was individually set to evoke an MEP of ~1 mV peak-to-peak at rest. Mean rMT, CS, and TS intensity values are provided for both young and older adults in Table 2. During the entire experiment, both TMS coils were continuously tracked with neuronavigation (Brainsight, Rogue Research Inc., Montreal, QC, Canada).

**Table 2:** Resting motor threshold (rMT), Conditioning Stimulus (CS) and Test Stimulus (TS) intensities per age group expressed as % of maximum stimulator output (Mean ± Standard Deviation).

	Younger	Older
rMT	39.19 ± 4.90	40.65 ± 6.27
CS intensity	47.03 ± 5.87	48.78 ± 7.52
TS intensity	58.57 ± 9.08	59.00 ± 9.72

#### Electromyographic Recording

Electromyographic (EMG) signals were collected from the left FDI muscle with self-adhesive 2-slot Bagnoli surface EMG sensors, connected to a Bagnoli-16 EMG system (Delsys Inc., Boston, MA, USA). The EMG signals were amplified (gain = 1,000), bandpass filtered (20–2,000 Hz) and 50/60 Hz noise was eliminated (Humbug, Quest Scientific, VN, Canada). MEP signals were stored on a laboratory computer for offline analysis.

### Experimental Protocol

**Figure 3:**
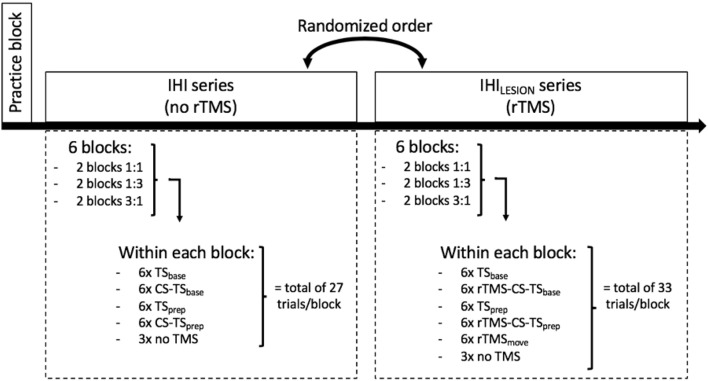
Schematic overview of the experimental protocol. After a practice block, the participant had to perform two series of six blocks of ~5 min per block, wherein he/she had to execute one of the three task conditions (1:1, 1:3 and 3:1). Each block contained two TMS conditions [TS and CS-TS for interhemispheric interaction (IHI) series, and TS and rTMS-CS-TS for IHI_LESION_ series], delivered either at baseline (“base”) or during the preparatory period (“prep”). Also, three trials without TMS were included in each block. In the IHI_LESION_ series, one extra rTMS condition was included during the actual bimanual movement (“rTMS_move_”).

A schematic and detailed overview of the experimental protocol is illustrated in Figure 3. First, participants were allowed to practice 12 trials of each task condition (1:1, 1:3, 3:1), to become familiar with the task variants. They were instructed to follow the white dot on the target line as accurately as possible, as well as to relax their hand muscles in between trials, which was monitored on-line with EMG. After the practice block, two series of six consecutive experimental blocks were performed. The six blocks of trials within each series consisted of two consecutive blocks of task condition 1:1, two consecutive blocks of 1:3, and two consecutive blocks of 3:1. The order of these three task conditions within each series was randomized across participants. Participants were informed before the start of each block which task condition they would perform next. In one series of six blocks, either TS and CS-TS trials (IHI series), or TS, rTMS_move_, and rTMS-CS-TS trials (IHI_LESION_ series) were assessed during the BTT. The order of these two series was pseudo-randomized across participants. In between all 12 experimental blocks, short breaks of ~5 min were provided. Each block had a duration of ~5 min. The triggers for TMS, BTT, and the auditory signal were controlled by Signal Software (version 6.0, Cambridge Electronic Design, UK).

#### Assessment of Interhemispheric Interactions (IHI) During Motor Preparation (IHI Series)

For the assessment of IHI (series without rTMS) during motor preparation, we used a protocol very similar to Fujiyama et al. (2016a); Fujiyama et al. (2016b). Over the two blocks of the same task condition within the IHI series, 12 TS and 12 CS-TS trials were collected either at baseline (“base”) or at the end of the preparation period (“prep”) to calculate IHI_base_ or IHI_prep_, respectively. More specifically, the timing of TS delivery (Figures 1C,D) was either at target line onset (i.e., the start of the preparatory period) or 50 ms before the imperative Go-signal, as inhibitory processes become stronger towards the end of motor preparation (Bestmann and Duque, 2016; Duque et al., 2017). Also, each task condition comprised six trials without TMS (i.e., no-TMS trials; three in each block) to obtain a measure of performance in the absence of TMS. Hence, each block of the IHI series consisted of a total of 27 trials, presented in a randomized order (Figure 3).

#### Assessment of the Neurophysiological and Behavioral Effect of DLPFC Suppression (IHI_LESION_ Series)

The six blocks in the IHI_LESION_ series were identical to the six blocks in the IHI series, except that the single CS on DLPFC was preceded by a “pulse train” of four consecutive pulses at 10 Hz (rTMS-CS-TS trials), to calculate IHI_LESION_ either at “base” or “prep” (i.e., IHI_LESION_ _base_ and IHI_LESION prep_, respectively). This protocol is well suited to study the neurophysiological effect of DLPFC suppression on DLPFC-M1 IHI (Hypothesis 1).

Furthermore, the direct effect of DLPFC suppression on motor performance (i.e., using TE and MI) was assessed (Hypothesis 2). More specifically, we made a distinction between the effect of DLPFC suppression during motor preparation and motor execution. For the latter, we used the rTMS_move_ condition. Each task condition involved 12 trials of the rTMS_move_ condition (i.e., six per block). Hence, for the IHI_LESION_ series, each block involved a total of 33 trials per block (Figure 3).

### Data Processing and Statistical Analyses

The statistical software R^1^ was used to perform all statistical analyses and to design all figures. For all statistical linear mixed models (lmerTest package, version 2.0-33), we included all 2-way and 3-way interaction effects. The original extensive models (i.e., with more than two independent variables) were then simplified by stepwise model building (i.e., removing stepwise non-significant interaction effects). For all statistical analyses, the normality of the residual data was checked via a normal quantile plot and homoscedasticity with residual plots. If model assumptions were violated, the outcome variable was transformed using the Box-Cox procedure (Box and Cox, 1964; MASS package, version 7.3-47). Significant main and interaction effects were further explored by Tukey HSD *post hoc* pairwise comparisons which control for multiple comparisons. The level of significance was set at α = 0.05.

#### Analysis of General BTT Performance (no rTMS)

Pure bimanual task performance was assessed based on the no-TMS trials. TE and MI were analyzed by a 2 (AGE: Young, Older) × 3 (TASK CONDITION: 1:1, 1:3 and 3:1) × 2 (SESSION: first, second) × 2 (SERIES: IHI, IHI_LESION_) linear mixed model. The factors AGE, TASK CONDITION, SESSION, and SERIES were included in the model as fixed effects, while SUBJECT was included as a random effect, to account for repeated measures within each subject.

#### IHI Modulation by rTMS—Hypothesis 1

Trials with TMS in which root mean square EMG in left FDI exceeded 20 μV (i.e., excessive muscle activity) during the 40 ms preceding the TS were discarded from the analysis.

We analyzed the modulation of DLPFC-M1 IHI during motor preparation, with or without rTMS. IHI modulation is expressed in IHI or IHI_LESION_ values, for the CS-TS trials or rTMS-CS-TS trials respectively (see above). IHI modulation was analyzed by using a 2 (AGE: Young, Older) × 2 (TIME: base, prep) × 3 (TASK CONDITION: 1:1, 1:3, 3:1) × 2 (SESSION: first, second) × 2 (TMS CONDITION: no rTMS, rTMS) linear mixed model, with AGE, TIME, TASK CONDITION, SESSION and TMS CONDITION as fixed effects and SUBJECT added as a random effect, to account for repeated measures within each subject.

#### Relationship Between Preparatory IHI Change Over Time and BTT Performance (no rTMS)

We explored the relationship between preparatory IHI change over time (i.e., without rTMS) and task performance, using Spearman’s rank correlations between IHI change scores on the one hand and TE and MI during the initial 1,500 ms of movement execution in no-TMS trials on the other hand, for both age groups. The IHI change score was defined as the ratio of IHI in the preparatory period expressed with respect to IHI at baseline (i.e., $\frac{IHI_{\text{prep}}}{IHI_{\text{base}}}$), assessed in the IHI series. Based on previous work (Fujiyama et al., 2016a), we applied a limited time window for calculating the task parameters TE and MI. Fujiyama et al. (2016a) argued that IHI change during motor preparation predicted the performance of only the first seconds of bimanual motor execution. In other words, for long-duration cyclical movements, it only makes sense to plan the initial period of movement execution rather than the whole trial.

#### Effect of DLPFC Suppression on BTT Performance—Hypothesis 2

The performance was assessed using TE for performance accuracy, and MI for performance stability. Note that for the behavioral performance effect, in contrast for the effect on IHI, we considered the CS on DLPFC to be part of the disrupting rTMS train, resulting in a train of five pulses over DLPFC at 10 Hz.

##### DLPFC Suppression in the Preparatory Period

**Figure 4:**
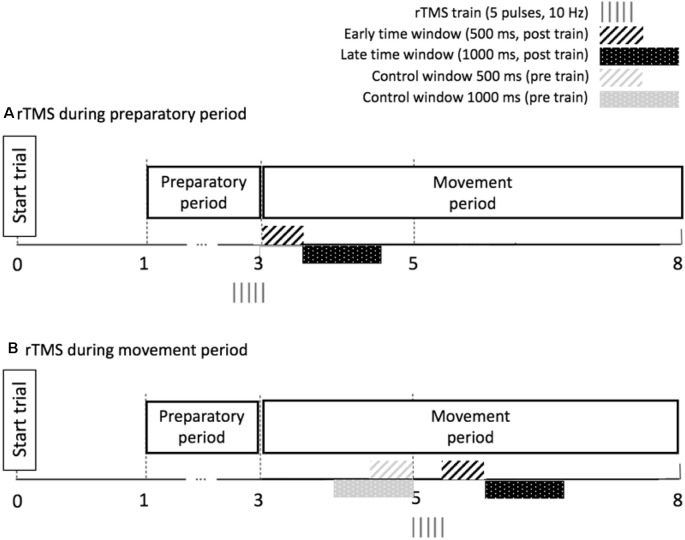
Time windows used for calculation of performance outcomes during rTMS in the preparatory period **(A)** and the movement period **(B)**. The duration of the early time window corresponds to the duration of the local transient lesion in the target region for rTMS. Note that for the movement period, we calculate performance ratios by dividing the performance after the pulse train by the performance before the pulse train.

As for the correlational analysis in “Relationship Between Preparatory IHI Change Over Time and BTT Performance (no rTMS)” section, we applied a limited time window during the initial period of movement execution for calculating the task parameters TE and MI. Specifically, we analyzed TE and MI in two subsequent time windows: the early time window and the late time window. For the early time window, the performance was investigated in the 500 ms epoch following the last pulse of the rTMS train, because the local effect of rTMS (10 Hz, five pulses) on underlying neural tissue is suggested to last 500 ms (Modugno et al., 2001; Duque et al., 2012). Additionally, we verified whether a behavioral effect could be observed in a subsequent late time window, corresponding to the 1,000 ms epoch following the early time window (Figure 4A). Performance measures were compared between rTMS-CS-TS trials and TS trials at “prep,” to control for the effect of a single TS immediately before movement onset. TE and MI were analyzed by a 2 (AGE: Young, Older) × 3 (TASK CONDITION: 1:1, 1:3 and 3:1) × 2 (SESSION: first, second) × 2 (TMS CONDITION: no rTMS, rTMS) linear mixed model, with AGE, TASK CONDITION, SESSION and TMS CONDITION as fixed effects and SUBJECT as a random effect. These analyses were performed for the early and late time window separately.

##### DLPFC Suppression in the Movement Period

The timing of the rTMS_move_ train (10 Hz, 5 pulses) was 2 s after the imperative Go-signal, and performance measures were calculated in the 500 ms epoch following the last pulse of the train (early time window) and in the subsequent 1,000 ms epoch (late time window). Because we were interested in whether short-train rTMS on DLPFC influenced the performance of ongoing bimanual movement, we quantified changes in TE and MI after the rTMS train by computing the following ratio: $\frac{Performance_{\text{post train}}}{Performance_{\text{pre train}}}$, for both the early (500 ms duration) and the late window (1,000 ms duration). The “pre-train” values were obtained by considering TE and MI in a control window of equal size (i.e., 500 or 1,000 ms, respectively) immediately before the pulse train onset (Figure 4B). This ratio was compared between rTMS_move_ trials and no-TMS trials, whereby the same time windows were chosen in the no-TMS trials as in the rTMS_move_trials. TE and MI ratios were analyzed by a 2 (AGE: Young, Older) × 3 (TASK CONDITION: 1:1, 1:3 and 3:1) × 2 (SESSION: first, second) × 2 (TMS CONDITION: no TMS, rTMS) linear mixed model, with AGE, TASK CONDITION, SESSION and TMS CONDITION as fixed effects and SUBJECT as a random effect.

#### Relationship Between rTMS-Induced Modulation of IHI and rTMS-Induced Modulation of BTT Performance in Older Adults

To explore whether the rTMS-induced gain in preparatory DLPFC-M1 disinhibition correlates with the rTMS-induced gain in performance accuracy captured in the late time window (see “Results” section), we performed an additional analysis. To do so, we defined a score for rTMS-induced modulation of IHI change over time [see “IHI Change Score” in the section “Relationship Between Preparatory IHI Change Over Time and BTT Performance (no rTMS)”] and for rTMS-induced modulation of performance accuracy, by calculating $\frac{IHI_{\text{LESION}}\text{change score}}{\text{IHI change score}}\left(=\frac{\frac{IHI_{\text{LESION, prep}}}{IHI_{\text{base}}}}{\frac{IHI_{\text{prep}}}{IHI_{\text{base}}}}\right)$ and $\frac{TE_{\text{rTMS}-\text{CS}-\text{TStrials,prep}}}{TE_{\text{TStrials,prep}}}$, respectively. TE was calculated in the late time window. Specifically, we used a Spearman’s rank correlation between these two rTMS-induced modulation scores in older adults.

#### Control Experiment

To investigate whether the rTMS effect over DLPFC on parameters of bimanual movement is region-specific, we ran a control experiment in which the right dorsal premotor cortex (PMd) was targeted. Previous TMS work indicated that the function of the right PMd is less task-specific than DLPFC or left PMd for bimanual motor control (Fujiyama et al., 2016a, b). Furthermore, current evidence suggests that PMd is less affected by aging than more prefrontal brain areas such as DLPFC (Raz et al., 1997, 2004; Seidler et al., 2010; Fujiyama et al., 2016b). The right PMd was individually localized on a 3D brain reconstruction (Brainsight, Rogue Research Inc, Montreal, QC, Canada), immediately anterior to the precentral sulcus and adjacent to the dorsal bank of the superior frontal sulcus (Davare et al., 2006; Duque et al., 2012; Fujiyama et al., 2016a, b). Mean Talairach coordinates are provided in Table 3. The protocol of this controlled experiment was highly similar to the main experiment, except for the following adjustments. First, since the PMd is less affected by age, only young adults were tested (*n* = 20; age range 18–33 years; mean ± SD = 22.85 ± 3.73; 11 females). These subjects were not part of the main experiment. Second, the stimulation intensity of the rTMS train on the right PMd was applied at an intensity of 110% of the individual rMT with an ISI of 8 ms between the rTMS train and TS on left M1. These stimulation parameters are best suited for IHIs of PMd with contralateral M1 (Kroeger et al., 2010; Hinder et al., 2012; Fujiyama et al., 2016a, b). Mean values for rMT and CS intensity are shown in Table 3.

**Table 3:** Control group characteristics: Mean PMd Talairach Coordinates—Resting Motor Threshold (rMT) and Conditioning Stimulus (CS) intensity, expressed as % of stimulator output (Mean ± Standard Deviation).

*x*	*y*	*z*	rMT	CS intensity
27.27 ± 3.33	−0.01 ± 3.62	58.05 ± 3.71	38.75 ± 8.05	42.63 ± 8.86

Similar to the main experiment, we defined two time windows (early window: 500 ms; and late window: 1,000 ms) for TE and MI calculations. The effect of PMd suppression in the preparatory period on TE and MI performance measures was compared between the average of 12 rTMS-CS-TS trials and TS trials at “prep”. TE and MI were analyzed by a 3 (TASK CONDITION: 1:1, 1:3 and 3:1) × 2 (TMS CONDITION: no rTMS, rTMS) linear mixed model, with TASK CONDITION and TMS CONDITION as fixed effects and SUBJECT included as a random effect.

For the effect of PMd suppression in the movement period (i.e., 2 s after the imperative Go-signal), we again assessed the change in TE and MI by calculating a $\frac{Performance_{\text{post train}}}{Performance_{\text{pre train}}}$ ratio and by comparing it between rTMS_move_trials and no-TMS trials. TE and MI ratios were analyzed by a 3 (TASK CONDITION: 1:1, 1:3 and 3:1) × 2 (TMS CONDITION: no TMS, rTMS) linear mixed model, with TASK CONDITION and TMS CONDITION as fixed effects and SUBJECT as a random effect.

## Results

### General BTT Performance

**Figure 5:**
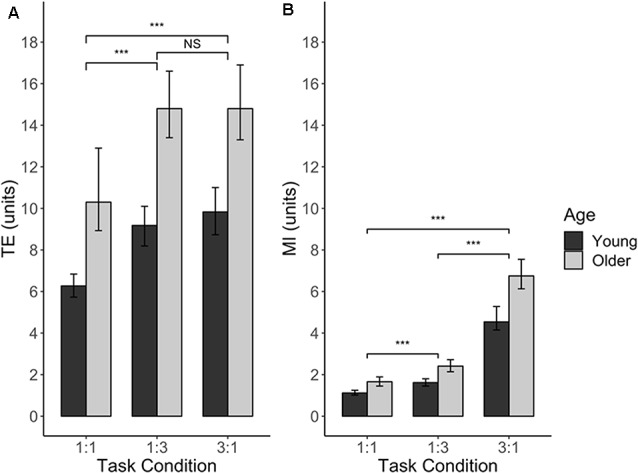
BTT performance without (r)TMS, expressed as Tracking Error **(A)** and Movement Instability **(B)**, plotted against different levels of TASK CONDITION and AGE. For both performance measures, there was a significant main effect of AGE, which was consistent over all the levels of TASK CONDITION (no significant AGE × TASK CONDITION interaction effects). Significance annotations are comparing differences between levels of TASK CONDITION, irrespective of AGE. Error bars indicate 95% CIs. Abbreviations: TE, Tracking Error; MI, Movement Instability; NS, Not Significant; ***p < 0.001.

For TE, three main effects were significant. For AGE (*F*_(1,39)_ = 21.92, *p* < 0.0001), young adults had a significantly lower TE (mean = 8.43 units) as compared to older adults (mean = 13.34 units), indicating better performance in young adults for this measure (*t*_(39)_ = −4.68, *p* < 0.0001). As expected, the main effect of TASK CONDITION (*F*_(2,202)_ = 66.70, *p* < 0.0001) indicated that the 1:1 condition (mean TE = 8.26 units) was better performed than the 1:3 (mean TE = 11.94 units) and 3:1 (mean TE = 12.28 units) conditions (*t*_(202)_ = −9.67, *p* < 0.0001 and *t*_(202)_ = −10.31, *p* < 0.0001, respectively). The performance between the latter two task conditions did not differ significantly (*t*_(202)_ = −0.64, *p* = 0.80). Lastly, the main effect of SESSION (*F*_(1,202)_ = 24.22, *p* < 0.0001) indicated that there was a learning effect during the experiment, with a lower TE in the second series of six blocks (mean = 9.90 units) compared to first series of six blocks (mean = 11.75 units; *t*_(202)_ = 4.92, *p* < 0.0001). None of the interaction effects were significant (all *p* > 0.18), nor was there an effect of SERIES (*F*_(1,196)_ = 0.40, *p* = 0.53), indicating that TE in no-TMS trials did not differ between the IHI series and the IHI_LESION_ series of the experiment. Figure 5A presents TE for each age group and each task condition.

MI was also found to depend on AGE (*F*_(1,39)_ = 21.83, *p* < 0.0001), with older adults showing higher instability (mean MI = 3.60 units) than younger adults (mean MI = 2.43 units). Moreover, MI was dependent on TASK CONDITION (*F*_(2,203)_ = 595.10, *p* < 0.0001). *Post hoc* analyses of this factor yielded that lowest instability was achieved in the 1:1 condition (mean MI = 1.38 units), which significantly differed from MI in the 1:3 condition (mean MI = 2.00 units) and 3:1 condition (mean MI = 5.62 units; *t*_(203)_ = −8.39, *p* < 0.0001 and *t*_(203)_ = −33.18, *p* < 0.0001, respectively). Moreover, the 3:1 condition yielded a significant higher instability than the 1:3 condition (*t*_(203)_ = −24.78, *p* < 0.0001). In contrast to the findings for TE, we did not observe an effect of SESSION for MI (*F*_(1,200)_ = 2.65, *p* = 0.11), indicating that there was no practice effect for this measure during the experiment. All interaction effects and the main effect of SERIES were not significant (all *p* > 0.25). Figure 5B presents MI for each age group and each task condition.

### IHI Modulation by rTMS—Hypothesis 1

For all trials with a TS, a total of 15.2% (11.4% in young adults; 19.2% in older adults) of trials were rejected due to excessive muscle activity recorded in the left FDI before the delivery of TS.

**Figure 6:**
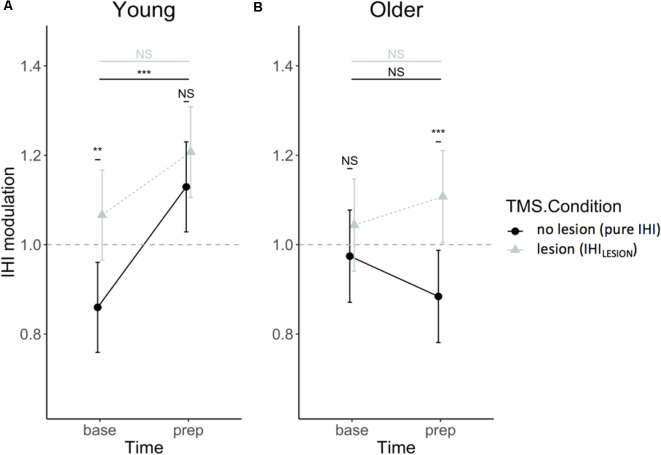
IHI modulation expressed relatively to single-pulse TMS, in IHI (dots) and IHI_LESION_ values (triangles), for young **(A)** and older **(B)** adults. Values above 1 indicate a facilitatory effect of the CS on DLPFC to contralateral M1. Values lower than 1 indicate an inhibitory effect of the CS on DLPFC to contralateral M1. Error bars indicate 95% CIs. Abbreviations: NS, Not Significant; **p < 0.01; ***p < 0.001.

DLPFC-M1 IHI modulation did not change throughout the experiment [i.e., no main effect of SESSION (*F*_(1, 432)_ = 0.71, *p* = 0.40)], and was not dependent on TASK CONDITION (*F*_(2,432)_ = 1.04, *p* = 0.35), nor were there significant interaction effects of SESSION and TASK CONDITION with other factors (all *p* > 0.39). The three-way AGE × TIME × TMS CONDITION interaction was significant (*F*_(1,432)_ = 5.55, *p* = 0.02), indicating that the interaction effect of TIME × TMS CONDITION on IHI modulation differed between age groups. The TIME × TMS CONDITION interaction effect was further explored for both age groups by *post hoc* Tukey HSD contrasts and is visually presented in Figure 6. The effect of TIME was only significant in young adults for IHI (*t*_(432)_ = −4.82, *p* < 0.0001) and not IHI_LESION_, which indicated significant DLPFC-M1 disinhibition during motor preparation, which was absent for older adults (*t*_(432)_ = 1.79, *p* = 0.28). A suppressing DLPFC lesion with rTMS had a significant effect on the DLPFC-M1 interaction in young adults at baseline (*t*_(432)_ = −3.84, *p* = 0.0008), but more importantly, in older adults at “prep” (*t*_(432)_ = −3.91, *p* = 0.0006). More specifically, in older adults, IHI at the end of the preparatory period was significantly modulated by DLPFC suppression, towards more disinhibition (Hypothesis 1; Figure 6).

### Relationship Between Preparatory IHI Change Over Time and BTT Performance

**Figure 7:**
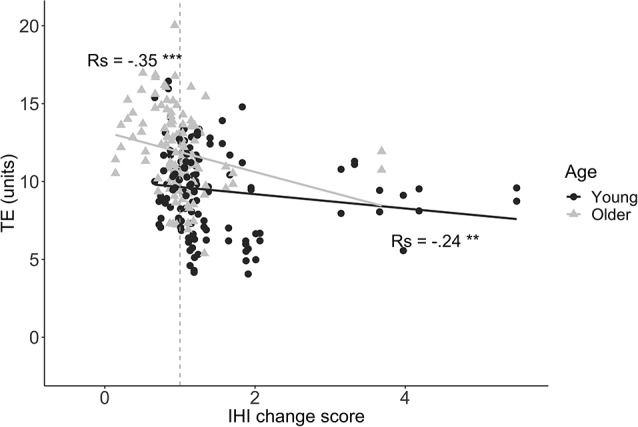
Tracking Error (TE) during the first 1.5 s of movement execution is plotted against IHI change scores $\left(\frac{IHI_{\text{prep}}}{IHI_{\text{base}}}\right)$, for young (dots) and older (triangles) adults. A negative correlation indicates that greater DLPFC-M1 IHI change during motor preparation is related to lower TE (i.e., better performance). The dashed line corresponds to an IHI change score of 1. Values below 1 indicate more inhibition in the late preparatory period as compared to baseline. Values above 1 indicate less inhibition (i.e., disinhibition) in the late preparatory period as compared to baseline. Abbreviations: TE, Tracking Error; Rs, Spearman’s rank correlation coefficient; **p < 0.01; ***p < 0.001.

We first explored the relationship between preparatory IHI change scores and task performance. A significant negative correlation between IHI change score and TE was found for both young (*r*_s_ = −0.24, *p* = 0.006) and older adults (*r*_s_ = −0.35, *p* < 0.0001), implying that a higher modulatory IHI capability during motor preparation was associated with lower error (Figure 7). For MI, there was no significant correlation with IHI change score for either the young (*r*_s_ = −0.1, *p* = 0.27) or older group (*r*_s_ = −0.03, *p* = 0.77).

### Effect of DLPFC Suppression on BTT Performance—Hypothesis 2

We restricted this part of the results section to the description of the main effect of and/or interaction effects with the variable TMS CONDITION on performance measures since this was our main variable of interest.

#### DLPFC Suppression in the Preparatory Period

In the early time window, TE was significantly affected by TMS CONDITION (*F*_(1,202)_ = 7.24, *p* = 0.008). Specifically, the error increased when DLPFC was suppressed with rTMS (mean TE = 5.55 units), compared to when it was not suppressed (mean TE = 5.31 units). All other interaction effects with TMS CONDITION were not significant (all *p* > 0.20). Interestingly, in the late time window the pattern reversed, as the error was lower in trials with DLPFC suppression (mean TE = 12.64 units) than without DLPFC suppression (mean TE = 13.25; *F*_(1,202)_ = 5.29, *p* = 0.02). Again, no interaction effects with TMS CONDITION were significant (all *p* > 0.29), indicating that the effect of TMS CONDITION on TE was not higher in older as compared to younger adults, nor was it different between all three task conditions.

**Figure 8:**
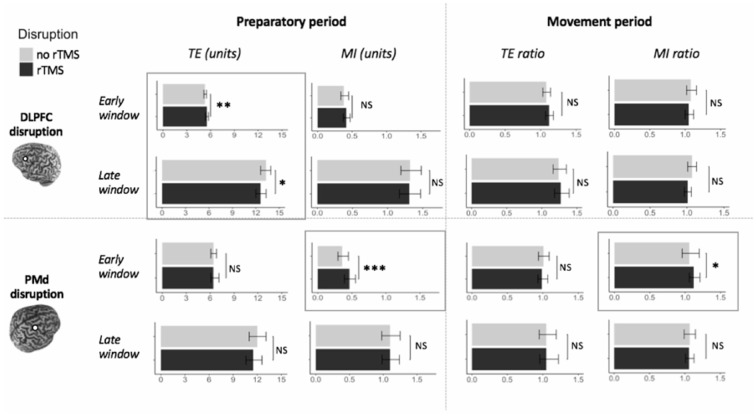
Effect of DLPFC and PMd suppression on actual bimanual movement. Upper panel: left DLPFC suppression; Lower panel: right PMd suppression; Left panel: suppression during movement preparation; Right panel: suppression during movement execution. Significant differences between the condition with disruptive rTMS (black bars) and the condition without disruptive rTMS (light gray bars) are marked by frames. Error bars indicate 95% CIs. Abbreviations: TE, Tracking Error; MI, Movement Instability; rTMS, repetitive TMS; NS, Not Significant; *p < 0.05; **p < 0.01; ***p < 0.001.

MI was not affected by TMS CONDITION in the early (*F*_(1,192)_ = 0.99, *p* = 0.32), nor in the late time window (*F*_(1,202)_ = 0.67, *p* = 0.42), nor were there any significant interaction effects of TMS CONDITION with other factors (all *p* > 0.39; Figure 8, left upper panel).

#### DLPFC Suppression in the Movement Period

TE ratio was not affected by DLPFC suppression during movement execution, in the early and late time window (*F*_(1,204)_ = 1.56, *p* = 0.21 and *F*_(1,200)_ = 0.27, *p* = 0.61, respectively), nor were there significant interaction effects with TMS CONDITION (all *p* > 0.39).

Similarly, MI ratio was not affected by TMS CONDITION in the early time window (*F*_(1,204)_ = 0.00, *p* = 0.99), nor in the late time window (*F*_(1,204)_ = 1.57, *p* = 0.21), and there were no significant interaction effects with this factor (all *p* > 0.20; Figure 8, right upper panel).

### Relationship Between rTMS-Induced Modulation of IHI and rTMS-Induced Modulation of BTT Performance in Older Adults

There was no correlation between rTMS-induced modulation scores for IHI change over time and rTMS-induced modulation scores for TE in the late time window (*r*_s_ = 0.11, *p* = 0.23). This indicated that, although DLPFC suppression during motor preparation increased DLPFC-M1 disinhibition in older adults, this was not related to the rTMS-induced gain in performance accuracy, as captured in the late time window.

### Control Experiment

A total of 0.071% of all trials were discarded for the performance (BTT) analyses due to technical problems. Again, we restricted this part of the results section to the description of the main effect of and/or interaction effects with the variable TMS CONDITION on performance measures.

#### PMd Suppression in the Preparatory Period

TE was not significantly affected by TMS CONDITION in the early and late time window (*F*_(1,99)_ = 0.04, *p* = 0.83 and *F*_(1,97)_ = 1.51, *p* = 0.22, respectively).

In contrast, in the early time window, MI was significantly worse when PMd was suppressed (mean MI = 0.46 units), as compared to when it was not suppressed (mean MI = 0.36 units; *F*_(1,97)_ = 11.43, *p* = 0.001). In the late time window, there was no significant difference in MI between the two conditions of TMS CONDITION (*F*_(1,97)_ = 0.04, *p* = 0.85).

The TMS CONDITION × TASK CONDITION interaction effect was not significant for both performance measures (all *p* > 0.09; Figure 8, left lower panel).

#### PMd Suppression in the Movement Period

TE ratio was not affected by PMd suppression in the early and late time window (*F*_(1,99)_ = 0.31, *p* = 0.58 and *F*_(1,99)_ = 0.15, *p* = 0.70, respectively).

On the contrary, MI ratio in the early time window was significantly worse when PMd was suppressed (mean MI ratio = 1.12), compared to when it was not (mean MI ratio = 1.06; *F*_(1,99)_ = 4.16, *p* = 0.04). In the late time window, there was no significant effect of TMS CONDITION on MI ratio (*F*_(1,99)_ = 0.00, *p* = 0.95).

There were no significant TMS CONDITION × TASK CONDITION interaction effects on both performance measures (all *p* > 0.46; Figure 8; right lower panel).

## Discussion

We used rTMS over left DLPFC in young and older adults to transiently suppress DLPFC function to go beyond the existing correlational evidence between left DLPFC—right M1 connectivity (or left DLPFC recruitment in general) and bimanual coordination by investigating its underlying causal mechanisms. Irrespective of age, more DLPFC-M1 disinhibition during motor preparation was related to lower TE, but in older adults, this disinhibition was reduced. Notably, suppressing DLPFC with rTMS increased DLPFC-M1 disinhibition in older adults. TE was initially (i.e., at the moment of lowered DLPFC excitability) worsened by rTMS, but this was immediately followed by a task accuracy improvement the next second. This behavioral rTMS effect was specific for the left DLPFC area, the preparatory period, and for TE, irrespective of age. Despite the positive effect of rTMS on both connectivity and behavior, the rTMS-induced gain in DLPFC-M1 disinhibition in older adults was not significantly related to the rTMS-induced gain in performance.

### Age-Related Changes in Preparatory Left DLPFC—right M1 IHI and the Modulatory Effect of rTMS

Our observations indicated a substantial left DLPFC—right M1 disinhibition during motor preparation in young adults, which was absent in older adults, likely resulting from a compromised white matter microstructural organization in the aging brain (Fujiyama et al., 2016b). Irrespective of age, more disinhibition was related to lower TE in the initial period of motor execution, in accordance with the findings of Fujiyama et al. (2016a,b). Results of our novel rTMS approach showed for the first time that transiently suppressing left DLPFC released interhemispheric left DLPFC—right M1 inhibition in older adults, resulting in restored disinhibition, corroborating Hypothesis 1. Both structural (Gooijers and Swinnen, 2014; Maes et al., 2017) and biochemical (Ding et al., 2016; Hermans et al., 2018; Pauwels et al., 2018) properties of the brain are prone to age-related neurodegeneration and are argued to affect cortical inhibitory functions (Seidler et al., 2010; Levin et al., 2014). Short-train rTMS possibly re-activated inhibitory cortical interneurons, leading to a transient, local suppression of DLPFC (Modugno et al., 2001; Duque et al., 2012). Our results suggest that this rTMS-induced suppression may be responsible for the release of DLPFC-M1 inhibition in the late preparatory period.

As higher DLPFC-M1 disinhibition predicted better performance accuracy (i.e., lower TE) in the first 1.5 s of movement execution and rTMS-induced DLPFC suppression during motor preparation increased this disinhibition, we predicted that rTMS would also result in an accuracy improvement in the initial movement period (Hypothesis 2). The current results of the behavioral effect of rTMS during preparation partly supported this hypothesis. Specifically, when accuracy was measured in a delayed time window after rTMS, a substantially better accuracy was observed as compared to trials without rTMS. In contrast, when accuracy was measured during the 500 ms following the rTMS train, it was worse than in trials without rTMS. Because during this early time window, the suppressing effect on DLPFC excitability is thought to be the strongest (Modugno et al., 2001; Duque et al., 2012), this finding might highlight the importance of intact left DLPFC functioning during bimanual motor preparation, which is in accordance with Santos Monteiro et al. (2017). Therefore, when predicting movement execution, it might be important to not only consider preparatory DLPFC-M1 connectivity but to consider DLPFC acting in a larger network, where connectivity with other regions might be crucial as well. Although this reasoning remains speculative, it is supported by an additional analysis, where we examined whether the rTMS-induced gain in preparatory DLPFC-M1 disinhibition in older adults was related to the rTMS-induced gain in accuracy, captured in the delayed time window. This would suggest a dominant role of this interaction over all other inter-regional connections with left DLPFC. However, the present study showed no relationship, suggesting that other inter-regional connections with left DLPFC, relevant for bimanual performance, might also be affected by rTMS and could explain the observed net effect on performance. These inter-regional pathways may include connections between the left and right DLPFC via callosal fibers (Sisti et al., 2012; Kim et al., 2014), ipsilateral DLPFC-M1 connections (Van de Vijver et al., 2014; Cao et al., 2018), cortico-subcortical circuits (Coxon et al., 2010; Levin et al., 2014; Bonifazi et al., 2018), connections with parietal regions (Madden et al., 2007; Van Impe et al., 2011), and with the cerebellum (Middleton and Strick, 2001; Dum and Strick, 2003; Kelly and Strick, 2003; Tanji and Hoshi, 2008). Future studies are warranted to investigate the exact contribution of each part of this network to the rTMS-induced performance effect.

### From Correlation to Causality: How Left DLPFC Regulates Bimanual Task Performance

Consistent with previous studies using similar tasks (Solesio-Jofre et al., 2014; Pauwels et al., 2015; Fujiyama et al., 2016b), younger adults showed better performance than older adults in terms of accuracy (TE) and stability (MI) of movement across all task conditions. Notably, our current findings showed that only TE was related to the magnitude of DLPFC-M1 disinhibition during bimanual motor preparation in both age groups. Accordingly, using disruptive rTMS, left DLPFC suppression during motor preparation directly caused changes in TE and not in MI, independent of age. TE reflects general performance, considering several parameters of bimanual coordination at once, such as accuracy for tracking the target dot, but also deviations of the subject’s track from the target line and movement speed. In contrast, MI captures the stability of the bimanual movement, regardless of speed or imposed target coordination pattern. A plausible explanation for the effect of DLPFC disruption on TE and not MI is that TE better captures the consequences of cognitively-guided preparation since the verbal instructions explicitly encouraged the participant to track the target dot as accurately as possible over the straight blue line, while performance stability (MI) was not explicitly emphasized. Preparatory attention and cognitive control of movement preparation are functions typically ascribed to DLPFC (Jueptner et al., 1997; Miller and Cohen, 2001; Tanji and Hoshi, 2008). DLPFC is considered essential for integrating sensory information with subsequent action in goal-directed behavior (Fuster, 1997; Lundy-Ekman, 2018). Regarding the current results, preparatory left DLPFC involvement likely constituted the association of visual signals with spatial motor acts through the retrieval of task-relevant information, to construct instructions for the actions to be planned (Hoshi and Tanji, 2004; Tanji and Hoshi, 2008). Moreover, since DLPFC is a core region in integrative action planning, visual signals might have been integrated with the prediction of sensory consequences of motor commands through connections with the cerebellum (Ebner and Pasalar, 2008; Tanji and Hoshi, 2008).

The observed contribution of left DLPFC to bimanual movements was shown to be region-specific. A control experiment targeting right PMd with rTMS showed that PMd suppression affected the stability of the movement (i.e., higher MI), but not accuracy (TE). Therefore, the role of right PMd is likely more focused on integrating two unimanual movements into a single control structure for both hands (Sadato et al., 1997; Toyokura et al., 1999; Kermadi et al., 2000; Debaere et al., 2004; Swinnen and Wenderoth, 2004).

In contrast, no effect of left DLPFC disruption was observed during movement on performance in both age groups. The role of DLPFC during cyclical bimanual movement execution has been documented more extensively in studies using conventional brain imaging techniques such as PET or fMRI (Fink et al., 1999; Remy et al., 2008; Goble et al., 2010; Beets et al., 2015; Santos Monteiro et al., 2017), mainly highlighting the cognitive control to monitor ongoing movement. However, causal inference deducted from these approaches is rather limited. Our study rather suggests that online monitoring of movement can be secured by other regions as well. On the other hand, the right PMd suppression during movement execution did worsen movement stability.

### Limitations and Future Directions

In the current study, rTMS was applied solely over the left DLPFC, which is why caution is needed when generalizing current findings to bilateral DLPFC. Although Fujiyama et al. (2016a,b), who used a highly similar study protocol, did not report any difference between left and right DLPFC in interhemispheric DLPFC-M1 interactions, there is evidence that the two regions have complementary functions during motor preparation (e.g., Vallesi et al., 2007; Beets et al., 2015). Thus, especially for the causal rTMS effect, the current findings are only applicable to the left DLPFC.

Next, with the current study protocol, it is not possible to define the exact reason why the rTMS-induced gain in interhemispheric DLPFC-M1 disinhibition and the rTMS-induced gain in performance accuracy in older adults were not significantly related. Nevertheless, this might suggest that both effects are driven through two different mechanisms. For example, the phenomenon of long intracortical inhibition (LICI), which might have been induced by the last two pulses of the rTMS train at 10 Hz (Sanger et al., 2001; Daskalakis et al., 2008; Vallence et al., 2014), could have already induced DLPFC-M1 disinhibition. In contrast, it is rather unlikely that only the last two pulses have induced the observed performance improvement in the delayed time window after the conditioning stimuli, since the local inhibition induced by two pulses is likely shorter (~150 ms; Daskalakis et al., 2008), as compared to the induced inhibition by four or five pulses at 10 Hz (~500–1,000 ms; Modugno et al., 2001; Siebner and Rothwell, 2003; Duque et al., 2012). Although a detailed understanding of the underlying neural mechanisms of the rTMS-induced effect falls beyond the goal of the current study, this should be further investigated in future studies.

Likewise, caution is needed when assigning a suppressing effect to short-train rTMS by the recruitment of local inhibitory mechanisms. For exploring physiological mechanisms of rTMS, previous work mainly focused on long-term plasticity mechanisms induced by extended application of rTMS (i.e., a higher number of pulses in rTMS-train and higher total doses; Di Lazzaro et al., 2010; Hoogendam et al., 2010; Baeken et al., 2017; Tang et al., 2017). In contrast, much less attention has been devoted to the effect of short-term transient lesions induced by short-train rTMS. Pharmacological or magnetic resonance spectroscopy (MRS) imaging techniques are needed to examine the role of gamma-aminobutyric acid (GABA), a principal inhibitory neurotransmitter, for explaining the local, transient suppressing effect of short-train rTMS (Cuypers et al., 2018; Pauwels et al., 2018). Insight in these mechanisms will be crucial to further explore the involvement of DLPFC in bimanual coordination.

Regarding the therapeutic implications of the current study, we do not recommend to use short-train rTMS over DLPFC, which was in this study exclusively intended to induce short transient effects for experimental reasons (i.e., inter-trial comparison, etc.). Rather, the current findings highlight the potential of inhibiting (left) DLPFC in the context of bimanual motor control in healthy older adults. Hence, we recommend that future research should focus on the use of less invasive methods (e.g., conventional cathodal tDCS; Nitsche et al., 2003; Zhu et al., 2015; Mosayebi Samani et al., 2019) that aim to induce long-term neuroplastic effects in DLPFC to inhibit its involvement during bimanual motor tasks and thereby improving performance in healthy older adults.

## Conclusion

This study showed that short-train rTMS over the left DLPFC can release right M1 inhibition during motor preparation in older adults and improve bimanual performance accuracy, but the direct link between these two rTMS-induced modulations remains unclear. We hypothesized that other inter-regional connections with DLPFC, besides the connectivity to M1, are also relevant for bimanual performance. Our results also suggest a causal link between left DLPFC and cognitively-guided bimanual motor preparation, while the online monitoring of ongoing movement is less crucially dependent on left DLPFC. In sum, our study highlights the potential of inhibiting (left) DLPFC in the context of bimanual motor control in healthy older adults.

## Data Availability Statement

The raw data supporting the conclusions of this article will be made available by the authors, without undue reservation, to any qualified researcher.

## Ethics Statement

The studies involving human participants were reviewed and approved by Commissie Medische Ethiek, UZ KU Leuven/Onderzoek, UZ Gasthuisberg Herestraat 49 B3000 Leuven (Belgium). The patients/participants provided their written informed consent to participate in this study.

## Author Contributions

The study design and protocol were developed by KC, SV, HF, and RM. All data were collected by SV, occasionally assisted by RM and OL. Data processing and analyses were run by SV and KD. SV has written the manuscript. All authors delivered important intellectual contributions to the interpretation of data, revised all drafts critically, and approved the submitted version.

## Conflict of Interest

The authors declare that the research was conducted in the absence of any commercial or financial relationships that could be construed as a potential conflict of interest.

## Footnotes

^1^http://www.r-project.org

## Abbreviations

CS: conditioning stimulus
DLPFC: dorsolateral prefrontal cortex
FDI: first dorsal interosseus
IHI: interhemispheric interaction
M1: primary motor cortex
MEP: motor-evoked potential
MI: movement instability
MRI: magnetic resonance imaging
PMd: dorsal premotor cortex
rMT: resting motor threshold
rTMS: repetitive transcranial magnetic stimulation
SD: standard deviation
TE: tracking error
TMS: transcranial magnetic stimulation
TS: test stimulus.
